# Ground Reaction Forces and Kinematics of Ski Jump Landing Using Wearable Sensors

**DOI:** 10.3390/s19092011

**Published:** 2019-04-29

**Authors:** Veronica Bessone, Johannes Petrat, Ansgar Schwirtz

**Affiliations:** 1 Department of Biomechanics in Sports, Faculty of Sport and Health Sciences, Technical University of Munich, 80992 Munich, Germany; johannes.petrat@tum.de (J.P.); ansgar.schwirtz@tum.de (A.S.); 2 Olympic Training Center of Bavaria, 80809 Munich, Germany

**Keywords:** landing, injury prevention, kinematics, kinetics, performance, winter sport, force insoles, inertial sensors, impact

## Abstract

In the past, technological issues limited research focused on ski jump landing. Today, thanks to the development of wearable sensors, it is possible to analyze the biomechanics of athletes without interfering with their movements. The aims of this study were twofold. Firstly, the quantification of the kinetic magnitude during landing is performed using wireless force insoles while 22 athletes jumped during summer training on the hill. In the second part, the insoles were combined with inertial motion units (IMUs) to determine the possible correlation between kinematics and kinetics during landing. The maximal normal ground reaction force (GRF_max_) ranged between 1.1 and 5.3 body weight per foot independently when landing using the telemark or parallel leg technique. The GRF_max_ and impulse were correlated with flying time (*p* < 0.001). The hip flexions/extensions and the knee and hip rotations of the telemark front leg correlated with GRF_max_ (*r* = 0.689, *p* = 0.040; *r* = −0.670, *p* = 0.048; *r* = 0.820, *p* = 0.007; respectively). The force insoles and their combination with IMUs resulted in promising setups to analyze landing biomechanics and to provide in-field feedback to the athletes, being quick to place and light, without limiting movement.

## 1. Introduction

Among ski jumping phases, landing has never been deeply scientifically investigated, being considered of minor interest both by researchers [1] and athletes [2]. However, landing and its preparation are important for performance and safety [1,3,4,5,6,7]. In fact, the athlete has to land using the telemark technique (step position) rather than with a parallel leg landing (squat position) in order to gain technical points from the judges for their overall performance score [8]. Concerning the safety aspect, in ski jumping, as in all jumping sports [9,10,11,12], injuries are frequent (around 21 for every 100 athletes) and involve the knee joint in 25 % of cases [13]. In particular, in jumping, a high ground reaction force (GRF) has been indicated as one of the main factors in non-contact anterior cruciate ligament (ACL) rupture [14], but also for other knee injuries [15,16]. In addition, when landing on an inclined surface, as in the case of the landing area of the ski jumping hill, the GRF and lower body kinematics vary [17]. Besides the landing height and the “heel first” landing strategy, GRF has been demonstrated to correlate with ankle, knee, hip and trunk flexion angles with the possibility of inducing knee injuries, as well as ACL rupture [15,18,19]. For example, subjects with ACL rupture had higher hip flexion during landing impact (50.1° versus 25.8°) [18]. Generally, the hip movement absorbs the upper body weight, while the ankle and knee joints absorb the GRF [19]. Besides the knee injury factor, we can speculate that a high GRF could influence balance during landing, with a possible consequent fall. Therefore, the quantification of the magnitude of the GRF, as well as the determination of the kinematics of the lower body during ski jump landing, could play an important role in injury prevention, providing feedback to the athletes and technical indications to coaches to optimize the landing gesture.

Besides the disinterest, one of the main reasons for the limited number of studies into landing was the difficulty of performing kinetic and kinematic analyses on the ski jumping hill, due to certain technical problems [1]: force insoles have the disadvantage of limiting the athlete movements due to the cables that connect the insoles with the receiver [4,20,21,22], while custom-made force-measuring bindings are relatively unsafe and need to be validated [23]. On the other hand, the integration of a force plate in the ski jumping hill table permits the measurement of the GRF only at take-off [24,25,26,27]. However, the development of wireless connections over the last years has permitted an evolution also in the sensors normally used during biomechanical studies. The introduction of wireless force insoles, connected by Bluetooth to a receiver or with an embedded battery and memory, could permit the performance of kinetic analyses of different sports, such as cross-country skiing and running [28,29], without interfering with athlete’s movements.

Concerning the kinematic analysis, inertial motion units (IMUs), with their easy-to-use, accurate and wide recording volume characteristics, overcome the problems related to the inaccuracy and the long post-processing and positioning time of the video camera set-ups [30].The IMUs have already shown their potential in ski jumping when positioned on the body [31,32,33] or only on the skis [34,35]. However, no studies have investigated the landing kinematics with the sensors positioned on the lower limbs, rather concentrating on the overall jump performance [31,32,33].

To the best of our knowledge, no studies have specifically investigated the impact force during ski jump landing and among different landing techniques. Our investigation is divided into two studies. The goal of the first study is to detect and quantify the magnitude of the force during telemark and parallel leg landing by means of loadsol wireless insoles (Novel GmbH, Munich, Germany). Secondly, an explorative study adding IMUs to the insole set up was performed in order to consider the possible correlations between kinematics and kinetics during landing.

The hypotheses were that longer jumps are correlated with higher impact forces, and that these forces are not equally distributed between both feet during parallel leg landing. Moreover, we hypothesized that the kinematics of the lower limbs influence the impact kinetics. Increasing the understanding of the landing biomechanics of ski jumping by means of wearable sensors could provide information on how the athlete should move in order to reduce the ground reaction force and, consequently, the possibility of injury. After determining this, a non-invasive set-up for landing analysis could be useful, not only for biomechanical research, but also for providing case-specific feedback to the athlete while training on the ski jumping hill.

## 2. Materials and Methods

The investigation is divided into two parts. In Study I, loadsol wireless insoles were used to detect the kinetics during ski jump landing in order to quantify the distribution, magnitude and impulse of the GRF among different subjects, different ski jumping hills and different landing techniques. In Study II, one subject was tested combining inertial sensors with the force insoles in order to detect possible correlations between kinetic and kinematic variables and to introduce a possible sensor combination to be utilized in further studies.

### 2.1. Study I

Twenty-two male ski jumpers and Nordic combiners (age: 17 ± 1 years old; weight: 65 ± 7 kg) competing at the National and International Junior level performing on the ski jumping hills HS100 in Oberhof (Germany), HS106 in Oberstdorf (Germany) and HS98 in Ramsau-am-Dachstein (Austria) during summer training conditions were studied. The total number of recorded jumps was 101: 37 in Oberhof, 38 in Oberstdorf and 26 in Ramsau-am-Dachstein. The athletes performed telemark or parallel leg landings, depending on their jump length and expertise. The participants were verbally informed in full about the nature of the study, signed a participation form and were allowed to withdraw at any point without giving a reason. The authors received the ethical agreement to the protocol of the study from the Dean of the Faculty of Sport and Health Science of the Technical University of Munich.

**Figure 1:**
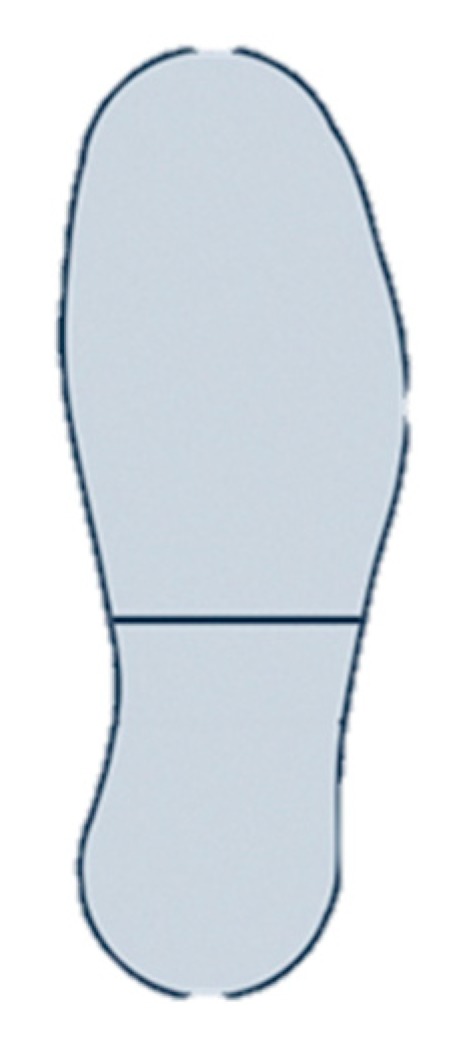
Detecting area division of the fore and rear foot part in loadsol force insole (adapted from [36]).

The athletes jumped wearing loadsol plantar force insoles, sampling at 100 Hz and were able to stop automatically after a certain time. The insoles were connected via Bluetooth to the app loadsol installed on an iPod (Apple, Cupertino, CA, USA), which worked as receiver and data logger and which was positioned on the arm of the athlete with a smartphone running case. The force insoles detected the fore/rear foot and overall normal ground reaction force; i.e., the force between the plantar side of the foot and the shoe [36] (Figure 1). The insoles have been previously validated [29,37,38,39]. Before each jump, the system was calibrated with the athlete’s body weight (BW) measured before the training using a body scale and including ski boots, helmet, gloves and ski suit. At the end of each jump, the athlete had to verbally report the kind of landing technique he performed.

**Figure 2:**
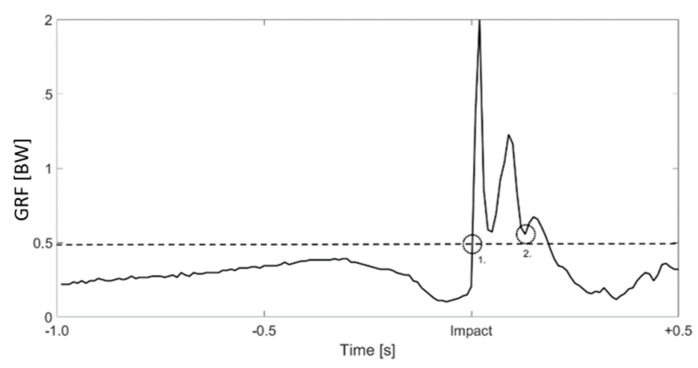
An example of the normal ground reaction force (GRF) (black line) from 1.0 s before the impact until 0.5 s after it. The dashed line represents 0.5 body weight (BW), used as threshold for the start of the landing (1.), while the end of the landing (2.) was defined as the minimum GRF after the second peak.

The recorded overall, rear and fore foot normal GRF were normalized on the BW and used as outcomes from the loadsol app, where the values are rounded in steps of 5 N. The maximum ground reaction force (GRF_max_) was the maximal GRF measured under each foot during the landing impact, while the impulse I (1) was calculated as

(1) $$I=\int_{t_{s}}^{t_{f}}\mathrm{GRF}dt$$

as reported in [23]. The start of the landing impact (t_s_) was defined as the first increase of normal GRF; i.e., when GRF was higher than 0.5 BW (Figure 2). The end of the landing (t_f_) coincided with the minimum of the signal after the second normal GRF peak after touchdown, corresponding with the end of the eccentric phase [23]. The difference between t_f_ and t_s_ defined the landing time (t_landing_).

The symmetry index (SI) (2) of the normal GRF_max_ and I between the two sides (indicated as variables x_L_ and x_R_ in (2)) was calculated according to [40] as

(2) $$\mathrm{SI}=\;\frac{\left|x_{L}-x_{R}\right|}{0.5*\left(x_{L}+x_{R}\right)}*100$$

A value of 0% indicates perfect symmetry, while a value equal or higher than 15% has been indicated to be a relevant asymmetry between the sides [41,42]. Finally, the distribution of the impulse between the front and rear foot was calculated as the ratio between I of the rear foot and the overall I, shown as percentage.

The duration of the flight phase (t_flight_) was considered to be the time between the end of take-off and t_s_, calculated for both feet and consequently averaged. The end of the take-off was considered when the normal GRF was smaller than 0.5 BW. Considering the wind condition to be stable during the entire data collection, we assumed that a longer t_flight_ corresponded to a longer jump.

The data processing was conducted using custom-written Matlab 2017a (Mathworks Inc., Natwick, MA, USA) codes.

### 2.2. Study II

One of the participants of Study I during the data collection on the ski jumping hill of Oberstdorf was additionally equipped with 11 inertial sensors aktos-t (myolution GmbH, Ratingen, Germany). The sensors were sampled at 143 Hz and were positioned on the skis, feet, shanks, thighs, pelvis, C7 vertebra and chest, placed under the ski suit and attached using medical tape. The procedure of calibration, drift correction and the use of inertial sensors is described in Appendix A. In total, nine jumps were collected using the combination IMUs and force insoles with the athlete always landing with telemark.

Three video cameras were added around the landing area to record the jump length. A light barrier at the take-off table, normally used during official competition, detected the take-off speed.

The kinematic data were firstly processed using the software iSen 3.08 (STT System, San Sebastian, Spain), which applied a low pass filter (Butterworth, second order, 10 Hz) to the raw data. Subsequently, a post-processing was performed using Matlab 2017a; if the hip, knee and trunk flexions/extensions showed negative values during the flight phase (i.e. not physiological extension) due to the offset created during the T-pose reference position [43], the angles were adjusted. In particular, the absolute value of the recorded minimum negative joint angle during the flight phase was considered as offset and added to the overall outcome. The flexion/extension, abduction/adduction and rotation of the knee and hip, the ankle dorsiflexion and abduction/adduction, and the orientation of the trunk (flexion, abduction and rotation) were analyzed at t_s_ after having post-synchronized the data based on the comparison of t_flight_ recorded by the insoles (calculated as explained in Study I) with the one recorded by the IMUs. The minimum ankle dorsiflexion after take-off was used to define the end of the take-off, while the end of the flight was defined as the first maximum ankle extension before the impact.

### 2.3. Statistical Analysis

Due to the high number of variables interfering with the movement and the different kinds of landing technique, each ski jump was considered as a specific case, even when performed by the same athlete. The mean and standard deviation (SD) are reported in order to show the magnitude of the detected variables in Study I. To determine the relationships between the kinematic and kinetic variables in Study I and II, Pearson correlations were calculated. The statistical analysis was performed using IBM SPSS Statistics (IBM Corp., Armonk, NY, USA). The criterion for statistical significance was set at *p* < 0.05.

## 3. Results

### 3.1. Study I

Of the 101 collected jumps, 56 were reported as telemark and 27 as parallel leg landing; the other 28 were not possible to classify, since the athlete reported to have not performed a distinct telemark or parallel leg landing. The average normal GRF_max_ per foot was 2.7 ± 0.9 BW (range: 1.1–5.3 BW; *n* = 101), respectively, of 2.6 ± 0.8 BW and 2.7 ± 0.9 BW (*n* = 56) for the telemark front and rear leg (max: 5.2 BW) and 2.7 ± 1.0 BW (*n* = 27) during parallel leg landing (max: 5.3 BW). The overall t_landing_ was 0.19 ± 0.05 s for both sides, while t_flight_ was 3.32 ± 0.29 s. The normal GRF_max_ and I correlated with t_flight_ (both *p* < 0.001), as reported in Table 1.

The SI indexes are reported in Table 2. The normal GRF_max_ during parallel leg landing was asymmetric (> 15% as according to [41,42]) in 81% of the cases, while I was asymmetric in 50% of the cases. During telemark landing, the normal GRF_max_ was asymmetric in 62% of the collected landings, while I in 68% of the cases.

**Figure 3:**
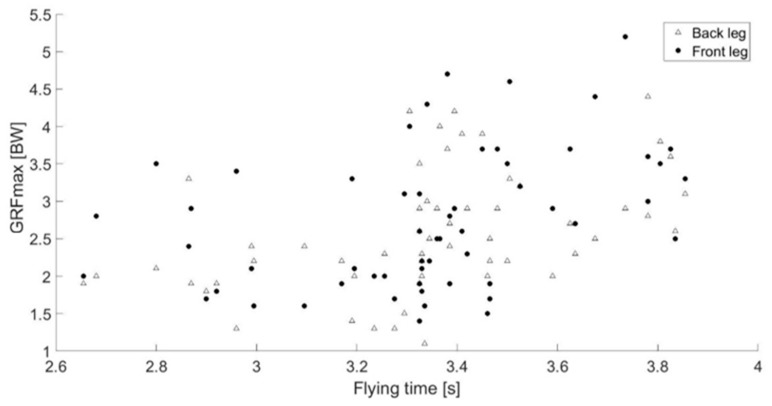
Normal GRF_max_ acting on the rear and front leg during telemark in relation to the flying time for 56 jumps.

The normal GRF_max_ distribution on the front (fl) and the back (bl) positioned leg in the telemark landing varied among the jumps and the t_flight_ (Figure 3).

Greater impulses correlated with greater normal GRF_max_ (left: *r* = 0.781; right: *r* = 0.776, both *p* < 0.001; *n* = 101). In Table 3, the correlations of the normal GRF_max_ and I with t_landing_ are shown in relation to the ski jumping hill where the data were collected.

### 3.2. Study II

During the nine jumps, the athlete equipped with IMUs and insoles always landed using telemark. The kinematic variables during landing impact were correlated with the opposite kinetic variable; for example, bl ankle flexion was correlated with the impulse of fl (*r* = −0.708, *p* = 0.033). The absolute values of the bl and fl hip flexion kept at t_s_ were correlated with the bl t_landing_ (*r* = −0.783, *p* = 0.013; *r* = −0.789, *p* = 0.011; respectively; *n* = 9). The fl GRF_max_ correlated with the fl knee rotation, fl hip flexion and bl hip rotation (*r* = 0.689, *p* = 0.040; *r* = −0.670, *p* = 0.048; *r* = 0.820, *p* = 0.007; respectively). No correlations were found between ankle dorsiflexion and torso angular movements and kinetic variables (*p* > 0.05).

The jump length recorded with the video cameras correlated with t_flight_ (*r* = 0.960, *p* < 0.001). The take-off speed (85.7 ± 0.7 km/h) did not correlate with any of the kinetic variables.

Values for t_flight_ calculated using the IMUs and using the insoles had an average difference of approximately 0.02 ± 0.02 s.

## 4. Discussion

In the study, wearable sensors were used to biomechanically analyze the ski jump landings. The main goal of the study was to determine the impact force and its distribution during different ski jump landing techniques by means of wireless plantar force insoles. Moreover, the detection of possible correlation between kinetics and kinematics during impact landing thanks to the introduction of the combination of IMUs and insoles was the goal of the explorative investigation of *Study II*. As assumed, the jump length was strongly correlated with t_flight_ (*r* = 0.960, *p* < 0.001). The post-synchronization for individuating t_s_ can be considered acceptable. The difference between the t_flight_ calculated using the IMUs and the force insoles was 0.02 ± 0.02 s, corresponding to 0.6% of the average t_flight_. Therefore, the calculation could be considered comparable. Not surprisingly, the primary finding was that a longer t_flight_ corresponded to a higher normal GRF_max_ due to the highest speed being reached and due to the flatter incline of the jumping hill [4]. Moreover, the normal GRF_max_ and the impulse were not symmetrically distributed between the two feet, independently from the landing technique. Finally, correlations between the hip and knee angles and the kinetic variables were found.

### 4.1. Study I

**Figure 4:**
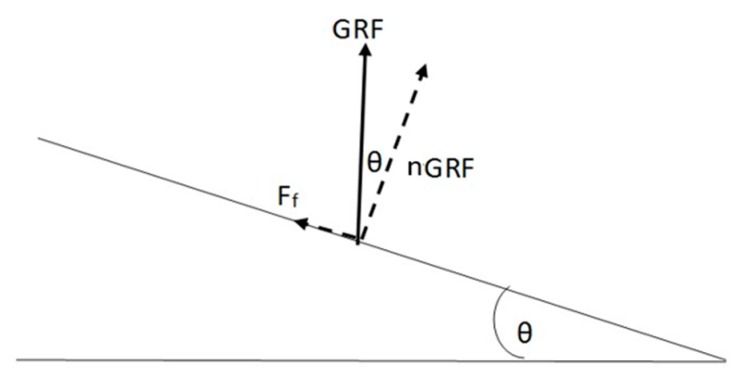
Overall GRF and its components (nGRF, normal GRF; F_f_: friction) related to the incline θ of the landing area.

The outcomes of the force insoles during the entire performance (Figure 2) were comparable with those in previous publications [1,21,35]. The normal GRF_max_ and the impulse were correlated with t_flight_ (Table 1). The range of the normal GRF_max_ per foot varied widely and, considering that the athletes landed with high speed, the magnitudes unexpectedly resulted to be relatively low in comparison to previous publications dealing with drop and countermovement jumps, which found a GRF_max_ per foot of 2.0 BW [45,46]. The reasons for the low magnitude could be related to technological, material and set-up problems. Firstly, previous publications disagreed about how the low sampling rate of the loadsol insoles could affect the capability of measuring impact: the groups of Burns [29] and of Seiberl [38] did not mention a limitation of the detection related to the low sampling rate, while the group of Peebles [37] stated that underestimation and overestimation biases of the impact force peaks were detected in single hop and stop jumps when using loadsol at 100 Hz (−0.46 BW, 0.36 BW, respectively) and at 200 Hz (0.37 BW, 0.35 BW, respectively). However, a difference of circa 0.4 BW would still represent a small contribution to the collected normal GRF_max_. Secondly, the ski jump boots are stiffer and with a more angulated shape in comparison, for example, to running shoes. When the athlete flexes his ankle during landing, he leans forward, with the shank pushing on the front part of the boot, carrying part of his BW. Therefore, in the data collection, part of the impact force could have been bypassed by the boot frame. Lastly, as reported by the insoles’ specifications [36], the collected impact represents only the normal component relative to the insoles’ surface. The overall GRF acting on the athlete is influenced by the incline (~35°) and, in particular, by the cosine of the incline (~0.82); therefore, the GRF collected by the insoles represents only circa 80% of the overall ground reaction force (Figure 4). Related to this, the correlations between kinetics and t_flight_ are more significant on the ski jumping hill of Oberhof (Table 3), where the incline of the landing area is the smallest (35°) and the cosine is therefore higher. This means that longer jumps on ski jumping hills with flatter landing areas lead to higher GRF.

The distribution of the normal GRF_max_ between the front and the back leg in telemark seemed to be case-specific (Figure 3). The overview of parallel leg and telemark landing SIs showed that the GRF_max_ and I are not equally distributed between the feet in both landing techniques in the majority of the collected jumps, although technically required by the International Ski Federation rules [8]. This could be explainable by a wider ski positioning leading to a one-side load with the possibility of ski edging [5] or by a possible different placement of the centre of mass during touchdown [46]. It can be assumed that the kinematics of the athlete play a role in the kinetic distribution and on the kinetics in general, as previously demonstrated [19]. To confirm this, thanks to the addition of the IMUs to the insoles’ set up in *Study II*, it has been possible to observe how the kinetic and kinematic variables were correlated. In some cases, due to the asymmetry of the telemark position, a kinetic variable of one side was correlated with a kinematic variable of the opposite side, as happened for the front leg hip flexion with the back leg t_landing_. However, an asymmetric position is not recommendable since it has been related to higher peak ACL forces in a simulation of jump landing in alpine skiing [47], as well as in studies on preventing ACL injuries [11,48].

The BW distribution between the front and the rear part of the foot seemed to be case-specific. Knowing this force distribution could be an important feedback for reducing injuries, since it has been shown how the ‘heels first’ landing technique results in higher vertical ground reaction force and smaller knee valgus and contraction in comparison to the ‘toes first’ approach when landing from a jump [15,19,49].

### 4.2. Study II

During the landing, athletes cannot modify the impulse acting on the force, since it is related to their body mass and velocity. However, the athlete can increase the t_landing_ acting on the hip, knee and ankle amplitudes, as occurs in gymnastics [49]. Respective to the joint movements of the subject analyzed in *Study II*, hip flexions/extensions, and knee and hip rotations of the telemark front leg were the movements that were mainly correlated with the normal GRF_max_. In the specific analysed case, in order to reduce the impulse, the athlete should try to land without rotating his front knee and hip joints, since the internal rotation of the knee is a risk factor for non-contact ACL injuries [49]. Moreover, in order to increase t_landing_, the analyzed athlete should keep the hips more extended at t_s_. However, since the lower body kinematics during landing vary according to gender and performance level, in normal jumping as well as in ski jumping [7,19,50], our findings might not be applicable to other athletes. Nonetheless, the study is the first to combine data from force insoles and IMUs positioned on the lower body and trunk, and it could provide perspectives for future research in the field. In general, an inertial sensor-based feedback has been shown to reduce the risk factors for ACL during drop jumps [51]. This application could also be effective for ski jump landing, giving suggestions to athletes about how they would need to perform in order to reduce the impact kinetics.

### 4.3. Limitations

Regarding the limitations of the studies, as previously mentioned, the low sample rate of the force insoles could have influenced the collected outcomes [35]. Therefore, during further studies, a higher sampling rate (200 Hz) of the loadsol insoles would be recommendable. The main limitation of *Study II* was the focus on only one subject. Due to the influence of the kinematics on the kinetics [9], further studies should be performed using a combination of inertial sensors and force insoles as proposed in the explorative investigation of *Study II*. In fact, a higher number of subjects would better describe the biomechanics of the landing, which vary among athletes according to gender, expertise and age [7]. Finally, a consideration for future research is that the researchers should pay particular attention when the athlete is getting dressed in their ski jumping suit, after having positioned the sensors on the skin. In fact, the suit could press on and move the sensors, resulting in incorrect outcomes.

## 5. Conclusions

The use of wireless force insoles to quantify the kinetic variables in ski jump landing could play an important role for injury prevention in this sport. The present study focused on the kinetics during landing impact in ski jumping, involving elite athletes during summer training and using wearable sensors. The combination of inertial sensors and force insoles did not interfere with the performance and resulted in non-invasive measurement according to the feeling of the jumpers. Therefore, from a practical point of view, the use of these wearable sensors during daily training could be effective for athletes, giving specific feedback on how they should move in order to reduce their vertical ground reaction force and impulse.

## Figures and Tables

**Table 1:** Correlations between the impulse and normal GRF_max_ of the left and right leg, and t_landing_ and t_flight_ for the 101 collected jumps.

	t_flight_	t_landing_
**F_max_**	left: *r* = 0.481 ***; right: *r* = 0.469 ***	
**I**	left: *r* = 0.552 ***; right: *r* = 0.538 ***	right: *r* = −0.263 **

^**^p < 0.01; *** *p* < 0.001

**Table 2:** Symmetry index (SI) of the normal GRF_max_ and impulse (I), and I distribution on the rear part of the foot during parallel leg and telemark landing (for the front (fl) and back (bl) positioned leg).

	Parallel Leg	Telemark
**Number of jumps**	26	56
**GRF_max_ SI between sides [%]**	24 ± 13	26 ± 21
**I SI between sides [%]**	15 ± 8	24 ± 17
**I distribution on the rear foot [%]**	56 ± 19	bl: 52 ± 25 (*n* = 55); fl: 48 ± 17

**Table 3:** Correlations between t_flight_, and normal GRF_max_ and impulse (I) acting on the left and right foot, in relation to the jumping hills where the data were collected. The landing area incline characterized the jumping hills [44].

	Ramsau am D.	Oberstdorf	Oberhof
**Incline of the landing area**	36°	35.5°	35°
**GRF_max_ (left)**	*r* = 0.517; *p* = 0.007	*r* = 0.363; *p* = 0.025	*r* = 0.597; *p* < 0.001
**GRF_max_ (right)**	*r* = 0.637; *p* < 0.001	*r* = 0.400; *p* = 0.013	*r* = 0.545; *p* < 0.001
**I (left)**	*r* = 0.342; *p* = 0.095	*r* = 0.554; *p* < 0.001	*r* = 0.651; *p* < 0.001
**I (right)**	*r* = 0.448; *p* = 0.022	*r* = 0.695; *p* < 0.001	*r* = 0.465; *p* = 0.004

## Appendix A

The inertial sensors aktos-t (myolution GmbH, Ratingen, Germany) used in *Study II* can be activated using a laptop and a receiver or by means of a remote control. In this last case, the sensors can store the collected data in their memory. The outcomes can be downloaded at the end of the data collection using the software iSen 3.08 (STT System, San Sebastian, Spain) and consequently analysed.

After having placed the sensors on the body of the athlete, a reference T-pose (posture with straight back, head looking forward and arms stretched to each side, forming a T-shape) needs to be performed [43]. The pose is performed before starting the data collection with the sensors connected by Bluetooth with a receiver and is required by the software iSen 3.08 to anatomically align each IMU to the body segment on which it is located [43]. This pose is the reference posture, where all joint angles are set to zero; therefore, all subsequent angles are recorded relative to it. During the data collection of *Study II*, the ski jumper found difficulties in keeping a steady T-pose with the leg fully extended while wearing the ski boots, since they are characterized by a specific stiffness and shape. Therefore, the athlete kept the T-pose position with the tips of the foot on a wooden bar. This stratagem was used to permit the athletes to have their knee and hip normally extended and in a normal standing position. The angle of the ankle was calculated with and without the bar in order to detect the difference between the two configurations. The difference was then considered during the data processing.

After the IMU calibration, the configuration of the sensor placement can be saved on the memory of the sensor. After this, the sensors can be switched on and off using the remote control. On the ski jumping hill, the sensors were switched on by an experimenter with the remote control while the athlete was preparing himself on the stairs beside the in-run. Before each jump, a T-pose was repeated with the tips of the ski boots on a wooden bar. This T-pose is required by the software iSen 3.08 to correct the drift accumulated during the data collection. After the jump, the sensors were switched off using a second remote control by an experimenter placed at the end of the landing area, with the athlete steady.
